# The Enduring Legacy of Randall Cohrs: A Meeting of the Minds in the Rocky Mountains

**DOI:** 10.3390/v14050915

**Published:** 2022-04-28

**Authors:** Charles Grose, Joel Rovnak, Ravi Mahalingam

**Affiliations:** 1 Virology Laboratory, Department of Pediatrics, University of Iowa, Iowa City, IA 52242, USA; 2 Department of Microbiology, Immunology and Pathology, Colorado State University, Fort Colins, CO 80523, USA; joel.rovnak@colostate.edu; 3 Department of Neurology, University of Colorado School of Medicine, Anschutz Medical Campus, Aurora, CO 80045, USA; ravi.mahalingam@cuanschutz.edu

Randall Cohrs established the Colorado Alphaherpesvirus Latency Society (CALS) in 2011. The main function of CALS was to support an annual meeting of investigators interested in herpesvirus latency and reactivation. Randall Cohrs died on 30 July 2021. In memory of Randy, we have assembled a Festschrift of articles written by virologists who attended CALS, as well as the Rocky Mountain Virology Association and the International Herpesvirus Workshop. Randy received his PhD degree from Southern Illinois University; the title of his doctoral thesis in the department of microbiology was “The molecular biology of *Herpesvirus sylvilagus*”. Thereafter, he moved to Colorado and later accepted a faculty position in the Department of Neurology at the University of Colorado School of Medicine. As a member of the Don Gilden laboratory, he devoted his career toward the study of transcription, latency and pathogenesis of varicella-zoster virus. In mid-career, he developed an interest in organizing scientific meetings. Based on knowledge acquired from that responsibility, he established CALS in 2011, specifically to bring together what he called a disjointed assemblage of virologists with shared interests in viral latency. CALS was a success. In this Festschrift, his friends and colleagues have submitted their contributions. The authors come from virology research centers in several countries, including the United States, the United Kingdom, the Netherlands, Germany, France, Switzerland, Israel, India, China and Japan. The viruses include, besides varicella-zoster virus and herpes simplex virus, cytomegalovirus, dengue virus, and coronavirus

## 1. Introduction to Festschrift

Randall Cohrs was the president of the Colorado Alphaherpesvirus Latency Society (CALS). The main purpose of CALS was to share scientific insights and foster collaborative research efforts among the gathered virologists. After his unexpected death in 2021, we have assembled a Festschrift in memory of Randy and his numerous achievements. Scientists who attended CALS, as well as Randy’s friends and colleagues from other virology meetings, were invited to contribute an article.

## 2. The Life of Randall Cohrs

Randy was a child of the American tall grass prairie. This massive ecosystem encompasses most of the states of North Dakota, South Dakota, Nebraska, Kansas, Iowa as well as sections of Illinois west and south of Chicago. The prairies formed 10,000 years ago and were once home to millions of bison. Long after the bison disappeared, Randy was raised in Illinois. The land is dotted with small villages and medium-sized towns, interspersed with thousands of acres of farmland. In turn, the farmland is crisscrossed by thousands of miles of train tracks. Even as a child, Randy always wanted to know more about how things worked. Randy enjoyed high school, where he enrolled in all the science classes; but he also won a letter for high achievement in cross-country running. Like all teenagers, he also had a variety of summer and after-school jobs, such as a short-order cook, a groundskeeper and even a cemetery caretaker. However, he never passed up a chance to go fishing. 

**Figure 1:**
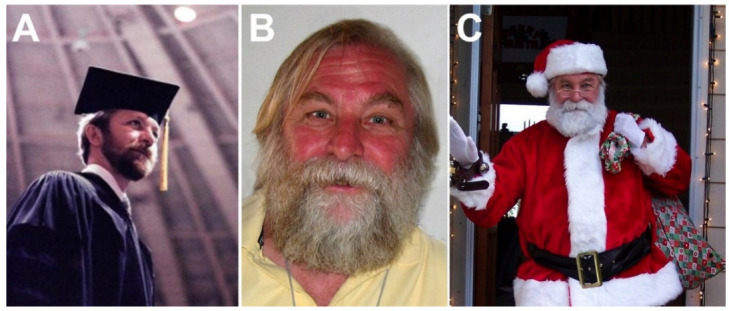
Photographs of Randall Cohrs during his career. (**A**). Graduation ceremony at Southern Illinois University in 1985. (**B**). Taking a break at a virology meeting in 2008. (**C**). Randy dressed as Santa Claus for a favorite children’s event in Lakewood, Colorado, in 2013.

After high school, he spent both his undergraduate and microbiology graduate college years at Southern Illinois University (SIU) in Carbondale, Illinois. He always described these years as fulfilling. He obtained his Ph.D. degree under the mentorship of Professor Hassan Rouhandeh on 20 March 1985. The title of his doctoral thesis was “The molecular biology of *Herpesvirus sylvilagus*”. Experimental infection with *Herpesvirus sylvilagus* produces clinical and histopathologic changes in its natural host, the cottontail rabbit (Sylvilagus genus), that are similar to those observed in humans acutely infected with Epstein–Barr virus (infectious mononucleosis). Randy’s most important Ph.D. research publication was a paper in the *Journal of Virology*, entitled *Herpesvirus sylvilagus: Polypeptides of virions and nucleocapsids* [1]. As expected, his thesis was one of the thickest on the shelf in the library (Figure 1). Randy was especially proud when colleagues at his former department asked him to give a special seminar for the SIU graduate students.

Randy moved to Colorado soon after graduation in order to be closer to his family who had moved there while Randy was in college. He accepted a post-doctoral position at the AMC Cancer Research Center in Lakewood, Colorado, with Professor Opendra Sharma, to study the role of vitamin E and the stability of RNA in cancer cells [2]. Soon afterward, he met Terri Carpenter; Randy and Terri were married on 3 January 1987, and they remained inseparable ever after. In 1989, when Don Gilden, Abbas Vafai and Ravi Mahalingam were looking for a RNA expert to investigate varicella zoster virus transcription, Randy Cohrs was their first choice. Randy was successfully recruited to a research-track faculty position in the Department of Neurology at the University of Colorado Health Sciences Center [3]. Therefore, except for the 4 years at the AMC Cancer Center, Randy would devote the rest of his scientific career to the study of herpesviruses (Figure 1). Randy was a very hard worker and was always focused on his next paper, grant or collaboration. His passion to understand basic biological processes was infectious. He also had insatiable thirst for other scientific fields such as astronomy, higher physics, and mathematics. Further, Randy easily engaged with the younger generation, infusing them with a curiosity for science. One of his frequently cited first-authored papers that drew considerable attention at virology meetings was a collaborative study funded by the National Aeronautics and Space Administration entitled *Asymptomatic reactivation and shed of infectious varicella zoster virus in astronauts* [4]. Another important first-authored paper entitled *Comparison of virus transcription during lytic infection of the Oka parental and vaccine strains of varicella-zoster virus* determined that viral IE62 (ICP4 homolog) was not the sole determinant of attenuation in the live varicella vaccine strain Oka [5]. Randy was also a member of the team that discovered the varicella-zoster virus latency-associated transcript [6]. Each of the authors of the current article were fortunate to have written one or more articles with Randy [7,8,9].

**Figure 2:**
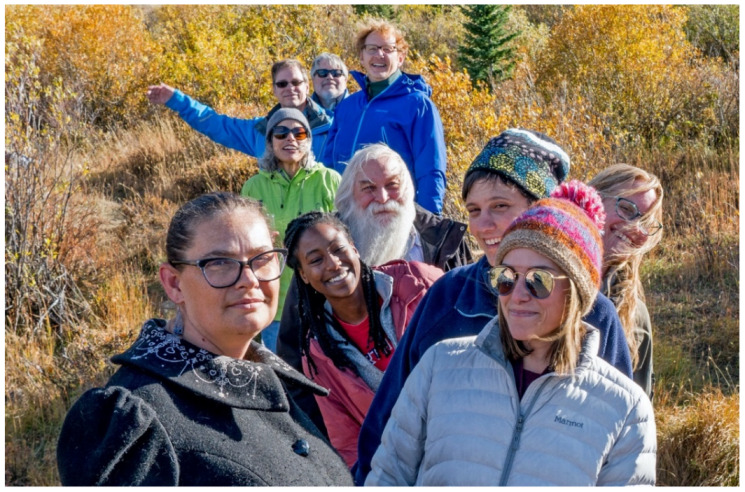
The Rocky Mountain Virology Association meeting in 2019. The photo shows several virologists at the meeting taking a hike in the mountains around the Colorado State University conference center during an afternoon recess. Randy, as usual, is at the center of an active discussion along the trail. Besides Randy, those in the picture include Laura Ashton, Carmen Ledesma-Feliciano, Christie Mayo, Amy MacNeill, Jasmine McCoy, Lee Fortunato, Sven Heinz, Joel Baines and Joel Rovnak. The meeting was held in late September when the golden leaves on the aspen trees are most beautiful.

At the seventh annual meeting of the Rocky Mountain Virology Club in 2007, Kathryn Holmes, from the University of Colorado, summoned Randy, Tony Schountz and Joel Rovnak from across the room to the business meeting, already in progress, and conferred upon them the administrative duties of procuring snacks and beverages for the next meeting. Two years later, Randy and Joel volunteered to run the meeting and Tony set up the website. At the same time, they established the Rocky Mountain Virology Association (RMVA), a 501(c)3 corporation. The RMVA could accept tax-free donations, but more importantly, RMVA could apply for support from the National Institutes of Health (NIH). From 2010 forward, the annual RMVA meeting held at the scenic Colorado State University Mountain Campus has been partially funded by the National Institute of Allergy and Infectious Diseases. With this NIH support, the organizers invited distinguished speakers from around the country. This change expanded the RMVA from a small regional meeting to one with national status. During these meetings, nothing excited Randy as much as making connections between investigators across a broad range of fields (Figure 2).

## 3. Colorado Alphaherpesvirus Latency Society

For a brief moment in this commentary, we introduce some pop-psychology to explain Randy’s enthusiasm for organizing virology meetings. There has been considerable research into the traits acquired by children in a family, based on their birth order. Almost everyone agrees that the first-born child is typically a high achiever. Randy was the middle child, having both an older and a younger brother. Randy had the archetypical persona of a middle child, including being a people-pleaser, thriving on friendships, having a large social circle, and playing the role of peacemaker. We believe that these characteristics gave him the ability, along with the valuable experience acquired from supervising the RMVA meeting, to establish the Colorado Alphaherpesvirus Latency Society (CALS) in 2011 (together with Don Gilden). We consider CALS to be one of the crowning achievements of Randy’s career. For nine consecutive years (2011–2019) until halted by the COVID-19 epidemic, CALS was held annually in mid-May at the Christiania Hotel in Vail, Colorado. CALS began as a meeting of 40 scientists interested in latency of alphaherpesviruses, but expanded to 70 when Don and Randy were able to secure rooms in the nearby Tivoli Lodge for additional participants. The rules were simple: submit an abstract, and give a 10 min talk followed by a 10 min discussion. The sessions over 2 days were divided into themes relevant to latency. Each CALS session had one guest speaker; the speaker at the 2019 CALS was the Nobel laureate Stanley Prusiner [10]. At night, the group meandered through the village to a restaurant. Randy always held his fireside chat on Friday evening after dinner. Randy carried out all these activities so many times with such a passion that his scientific influence was felt well beyond the varicella and herpes world.

## 4. Anecdotes

Each author will present below an anecdote about Randy, which provides more insight into his persona both inside and outside the world of science.

### 4.1. From Charles Grose

Both Randy and I lived similar childhoods. We talked about trains, not model toy trains but real trains. Both of us were raised on the prairie, where trainspotting was a popular game for children. When a train was first seen or when a train whistle was heard miles away, he walked to the tracks to be only a few feet away when the 200-ton locomotive rumbled past him. The engineers always waved. As his first scientific experiment, he placed penny coins on the tracks and waited to see which became the widest after being crushed by the locomotive. Randy’s favorite railroad was the Soo line because of its weird name, a phonetic abbreviation of the Minneapolis, Saint Paul and Sault Sainte Marie railroad (French word Sault = Soo in English). When Randy moved to Colorado, he switched his allegiance to the scenic Denver and Rio Grande Railroad. Joel later told me that Randy liked Joel’s office because Joel could hear sounds of passing trains from his office.

### 4.2. From Ravi Mahalingam

In the late 1970s, both Randy and I were graduate students at SIU in microbiology and chemistry, respectively. Later, we were colleagues working together on varicella-zoster virus latency and reactivation in the same department at the University of Colorado School of Medicine. Having had the privilege of interacting closely with Randy for the past 4 decades, I noticed that the five words that Randy used most often was “how can I help you“. Whether it was a person that he met in the street for the first time or a longtime acquaintance, Randy was always finding ways to help them when there was a need. I can never forget how Randy carried my hand carriage with the broken wheel up the hill to my room in Regensburg, Germany, and when he rolled my 3-year-old son in his stroller throughout the museums in Washington, DC. Additionally, Randy had a knack of making friends independent of language and cultural barriers. I remember him spending more than an hour with a total stranger (who did not know English), whom he bumped into during his visit to India. Randy’s passion for life and curiosity to learn will be missed.

### 4.3. From Joel Rovnak

Randy and I spent hours brain-storming transcription regulation and working out chromatin immune precipitation protocols. He was an early proponent of next-generation sequencing and did everything he could to promote the core sequencing capacity at the University of Colorado School of Medicine and to support investigators with the skills to analyze sequence data. To that end, we spent a lot of time selecting guest speakers from the sequencing and transcription fields for our virology meetings, more than one of whom lamented that they were not virologists and questioned why were we inviting them to a virology meeting. They came to the meetings and came back again and again. One of Randy’s greatest joys was introducing people who would never meet otherwise. He knew that they needed to meet and just had not realized it yet.

## 5. The Final Day

On Friday, 30 July 2021, Randy was working late in the laboratory as usual. Just before he left, he arranged meetings the next day with students and colleagues to practice talks for a meeting on Monday. While driving home he must have known something was amiss with his health, because he took care to pull over to the side of the highway out of traffic, one last act of consideration for others. The impact of his death extends far and wide. Randy is buried in Golden, Colorado, at the historic Golden Cemetery, Section 238. Graciously, the journal *VIRUSES* has given his friends and colleagues an opportunity to assemble a Festschrift by which to recognize and remember the legacy of Randy Cohrs.
